# Online Adaptive Prediction of Human Motion Intention Based on sEMG

**DOI:** 10.3390/s21082882

**Published:** 2021-04-20

**Authors:** Zhen Ding, Chifu Yang, Zhipeng Wang, Xunfeng Yin, Feng Jiang

**Affiliations:** 1 School of Mechatronics Engineering, Harbin Institute of Technology, Harbin 150000, China; 16B908060@stu.hit.edu.cn (Z.D.); cfyang@hit.edu.cn (C.Y.); 19B908078@stu.hit.edu.cn (Z.W.); 19S108220@stu.hit.edu.cn (X.Y.); 2 School of Computer Science and Technology, Harbin Institute of Technology, Harbin 150000, China

**Keywords:** motion intention prediction, sEMG, convolution autoencoder, online adaptation, wearable sensors

## Abstract

Accurate and reliable motion intention perception and prediction are keys to the exoskeleton control system. In this paper, a motion intention prediction algorithm based on sEMG signal is proposed to predict joint angle and heel strike time in advance. To ensure the accuracy and reliability of the prediction algorithm, the proposed method designs the sEMG feature extraction network and the online adaptation network. The feature extraction utilizes the convolution autoencoder network combined with muscle synergy characteristics to get the high-compression sEMG feature to aid motion prediction. The adaptation network ensures the proposed prediction method can still maintain a certain prediction accuracy even the sEMG signals distribution changes by adjusting some parameters of the feature extraction network and the prediction network online. Ten subjects were recruited to collect surface EMG data from nine muscles on the treadmill. The proposed prediction algorithm can predict the knee angle 101.25 ms in advance with 2.36 degrees accuracy. The proposed prediction algorithm also can predict the occurrence time of initial contact 236±9 ms in advance. Meanwhile, the proposed feature extraction method can achieve 90.71±3.42% accuracy of sEMG reconstruction and can guarantee 73.70±5.01% accuracy even when the distribution of sEMG is changed without any adjustment. The online adaptation network enhances the accuracy of sEMG reconstruction of CAE to 87.65±3.83% and decreases the angle prediction error from $4.03^{\circ }$ to $2.36^{\circ }$. The proposed method achieves effective motion prediction in advance and alleviates the influence caused by the non-stationary of sEMG.

## 1. Introduction

The exoskeleton control systems are usually divided into high-level, middle-level, and low-level control systems [1]. The high-level control system is used to monitor human movement and recognize human intention. The middle-level control converts the human intention to the control trajectory of the exoskeleton. The low-level control system is usually adopted to achieve the precise control of the actuator. The efficacy of the exoskeleton is determined mainly by the accuracy and timeliness of human intention recognition. In order to improve motion recognition accuracy, various robotics applications have adopted surface electromyography (sEMG), which contains movement information. Other than the high accuracy issue, two more crucial challenges of current sEMG-based human intention recognition methods are the time delay and the sEMG non-stationary.

Due to the sensor delay, the movement is captured after it actually happens. Meanwhile, even if the movement intention is correctly recognized, considering the challenge of the process of those complex signals and the delay of the control system and the actuator, it is still difficult to have an exact match between the exoskeleton and the human movement. The unexpected interaction between the exoskeleton and human leads to poor assistance effect.

In the motion intention perception field, many types of research focus on real-time intention detection. These studies generally consider a certain amount of delay to be acceptable [1,2,3,4,5,6,7]. For example, Hudgins et al. [4] stated that a myoelectric hand control system’s response time should be less than 300 ms so that the user operates the hand without perceiving a time delay. Young et al. [8] used 300 ms windows of sEMG signals combining with motion signals to obtain high classification accuracy in motion mode recognition. Simon et al. [9] achieved higher classification accuracy by increasing latency. On the other hand, there are some studies considering the balance between delay and accuracy. This is because algorithm delay and mechanical system delay will make the actual motion of the actuator unable to track the designed trajectory (force or motion). In order to alleviate the time delay, HeHuang [10] proposed a locomotion-mode prediction method during mode transitions. By predicting the movement mode, the artificial knee controller can switch the control parameter (such as impedance) in time. The prosthesis can perform seamless and safe locomotion-mode transitions. Pew et al. [11] proposed a predictive method that uses IMU and sEMG signals to predict turn intention 400 ms in advance with more than 95% accuracy. However, those prediction algorithms are only aimed at the transition of motion pattern, and it cannot predict the motion in advance in the stable state such as level walking. Meanwhile, most of those methods label the sEMG window using the label of data in the middle of the window to ensure classification accuracy, which delays the intention algorithm half time window.

sEMG signals have been proved to improve the accuracy of intention prediction in [8,12,13,14]. However, the non-stationary of sEMG signal increases the uncertainty of the motion recognition algorithm [15,16]. Non-stationary on muscle refers to changes in muscle characteristics due to individual difference, muscle fatigue, and human adaptation [3,17,18,19,20]. In general, individual differences manifest as the mutation of sEMG distribution between individuals caused by the anatomical differences between the subject. Muscle fatigue and human adaptation are shown as slow changes in sEMG distribution over time. Muscle fatigue is mainly caused by central and peripheral factors. Human self-adaptation is the exploration and exploitation of the human body when the external environment changes (such as external assistive of the exoskeleton). The non-stationary nature of signals leads to a decrease in the accuracy and reliability of the recognition algorithm.

In order to mitigate non-stationary characteristics and ensure the reliability of the sEMG signal during the application, some research focuses on feature engineering, which improves the robustness of the algorithm through data-driven automatic feature extraction [21,22,23,24,25]. Spanias et al. [26] used a log-likelihood metric to detect the sEMG disturbances and then to decide whether to use it or not. When sEMG contained disturbances, the classifier detected those disturbances and disregarded sEMG data. The reliability of the recognition algorithm can be guaranteed by giving up those unqualified sEMG signals. However, from the accuracy of the algorithm, it is inexpedient to discard any existing signal. Despite changes in sEMG distribution, it still contains motor information. Du Yu et al. [27] introduce the Adaptive Batch Normalization (AdaBN) transfer learning algorithm into sEMG signal processing. By online adjusting the mean and variance of the Batch Normalization (BN) layer, the AdaBN algorithm can improve the prediction accuracy to some extent. The most crucial feature of AdaBN is its low computational load and short optimization time. However, the AdaBN’s improvement in accuracy is limited, especially for conventional low-density electrodes, which cannot be applied in practice.

The goal of this paper was to accurately and reliably predict the future motion of the lower limb. Our work consists of three major contributions.

1. This paper proposes a joint angle and special events prediction method based on sEMG signals. From the exoskeleton perspective, obtaining the motion intention before it occurs can help the controller relieve the algorithm delay, mechanical delay, and better tracking behavior.
2. This paper presented an efficient sEMG signal feature extraction method. The proposed method based on convolutional autoencoder network combining with muscle synergy trick can obtain the reconstructible and compressed sEMG feature.
3. We embedded a deep adaptation mechanism into the motion prediction algorithm. The proposed adaptation method can effectively alleviate the non-stationary sEMG signal and ensure the reliability of the prediction.

## 2. Method

### 2.1. Participants and Measurements

#### 2.1.1. Participants

A total of 10 subjects ranging in age from 24 to 28 years participated in the experiments. None of the participants had any motor dysfunction. Each subject was instructed to walk on the treadmill.

The study was conducted in accordance with the Declaration of Helsinki and was approved by Chinese Ethics Committee of Registering Clinical Trials (ChiECRCT20200319). Written informed consent was obtained from all subjects.

#### 2.1.2. Acquisition

In this study, we adopted the IM sensors of the Delsys Trigno surface EMG system to collect the sEMG and inertial measurement unit (IMU) signals. A total of 11 IM sensors (nine for sEMG, two for IMU) were used to monitor the lower limb’s status.

**Figure 1:**
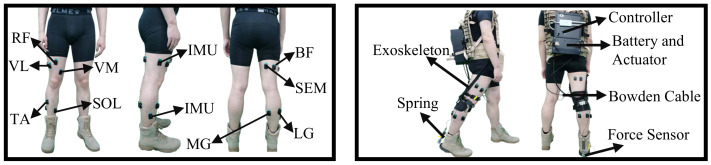
A sketch of the sensors setup and the exoskeleton system.

These nine muscles are rectus femoris (RF), vastus medial (VM), vastus lateralis (VL), tibialis anterior (TA), soleus (SOL), semitendinosus (SEM), biceps femoris (BF), medial gastrocnemius (MG), and lateral gastrocnemius (LG). Note that the locations for electrode placements were approximate and guided by palpation and EMG recordings when the subjects were instructed to perform hip and knee flexion/extension and adduction/abduction. The skin for electrode placements was cleaned with rubbing alcohol prior to electrode placement. Two IM sensors mounted on the thigh and shank were implemented to obtain the limb’s three-axis acceleration and angular velocity. The IMU placement positions were selected to keep away from the main muscle group avoiding the shake caused by the muscle, as Figure 1 shown. The sampling rate of sEMG and IMU signals were 1111.111 Hz and 148.148 Hz (sampling intervals are 0.9 and 6.75 ms), respectively. The two-channel Trigno FSR sensor was adopted to estimate the special event heel strike. The sampling rate was 148.148 Hz. All signals were collected synchronously. Once all the sensors were placed properly, the participants were asked to stand still on the treadmill. After running the acquisition software on the laptop, the participants were advised to walk at a speed of around 4.5 km/h. The final walking speed was determined by each participant. The mean and standard of walking speed throughout the walk for 10 subjects is 4.31±0.174 km/h. The total duration for each participant to walk on the treadmill was 42 min.

To initially verify the influence of the exoskeleton on the prediction algorithm, the exoskeleton experiment was conducted in this paper. As Figure 1 shown, the adopted ankle exoskeleton system consists of the controller, actuator, and exoskeleton structure. The exoskeleton can provide the assistive force in plantarflexion. The exoskeleton has three working positions: initial, assistive position, and zero force. The three working positions respectively correspond to the position of the motor when the force sensor is 10 N, 100 N, and 0 N. The initial, assistive, and zero force position respectively work during 0∼30%, 30∼65%, and 65∼100% gait phase of norm walking. The controller consists of the iterative algorithm. During the assistive phase, the controller will alter the assistive position if the maximum force is less or greater than 100 N. During the zero-force phase, the controller will alter the zero-force position if the force is greater than 0 N. Four subjects participated in the exoskeleton experiment. After the placement of sensors and exoskeleton, the participants were asked to stand still. The exoskeleton started pretightening until reaching the initial position. Then, the participants began to walk, and the exoskeleton was turned off. The participants walked for 21 min without any assistance from the exoskeleton. Then, the assistive switch was turned on. The participants walked for 21 min with assistance from the exoskeleton.

#### 2.1.3. Data Preprocessing

The first and last minute of data for each participant were discarded due to the instability of movement during acceleration and deceleration. The three-axis acceleration and angular velocity of two IMUs were utilized to estimate the knee angle with the angle estimation method [28]. Then, the acceleration and angular velocity of IMU and joint angle were converted into motion sequences with 13 samples (87.75 ms). The motion sequences were overlapped and the interval between two adjacent motion sequences was 2 samples (13.5 ms).

The sEMG signals were segmented using a sliding window with a length of 200 sEMG samples (180 ms). The increment of the sliding windows was set to 15 samples (13.5 ms) according to the least common multiple of sEMG and IMU sampling interval. Under this operation, the motion sequence and sEMG windows were synchronized to each other. Then, the sEMG windows from different muscles at the same moment were combined into a matrix with the size of 9×200 named sEMG image, in the order of RF, VM, VL, TA, SOL, SEM, BF, MG, and LG. [24,29,30] The horizontal axis of the sEMG image represents the different sEMG channels. The vertical axis of sEMG image represents the different sEMG samples. The FSR signal of heel was used to obtain the special event heel strike. For each IMU sample, the time to the next heel strike was calculated.

This work utilized different data to label the sEMG images and motion sequences for different types of tasks. For the special event prediction task, the sEMG image and motion sequence were labeled using the time to heel strike. For the joint angle prediction, each pair of motion sequence and sEMG image was labeled using the joint angle of the future. Four groups of angle label were prepared for each pair of sEMG image and motion sequence: joint angle after 6.75, 33.75, 67.5, 101.25 ms.

### 2.2. Motion Prediction and Domain Adaptation

**Figure 2:**
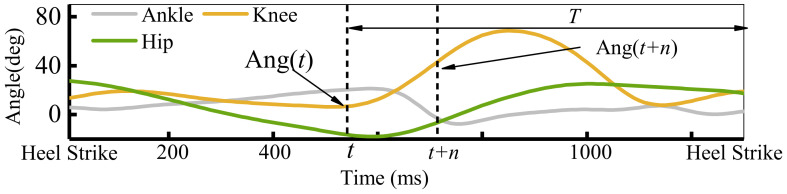
The lower limb joint angle and special event for a gait.

Human motion intentions usually refer to joint angle, motion mode, gait phase, and special event points such as heel strike in exoskeleton control systems. Especially for joint angle and heel strike event, they are often used to map the joint torque or determine the initiate of the gait phase. Figure 2 shows the lower limb joint angles and heel strike event points during a complete gait phase. A complete gait phase starts with the heel strike and ends with the heel strike of the same leg. The motion prediction means not only detecting the movements as it happens but also predicting the movements some time ahead. As presented in Figure 2, the joint angle prediction means the algorithm takes the input of Ang(t) and outputs the future angle Ang(t + n), where n is the advance time. The special event prediction is to predict the arrival time T of the next heel strike with the same leg.

The proposed sEMG-based motion prediction algorithm consists of the prediction network, the feature extraction network and the adaptation network. Each part is responsible for different functions but is closely related.

The prediction network based on LSTM is applied to predict the future motion information using the motion sequence and sEMG features. However, only relying on the prediction network cannot guarantee the accuracy and reliability of the results. Considering the non-stationary characteristics of sEMG signals, this paper devises the feature extraction network and the online adaptation network to handle the sEMG signals.

The feature extraction network utilizes the convolutional autoencoder neural network to extract the compressive and robust features from the sEMG signals. In general, surface EMG signals are thought 100 ms advanced occur than the movement of the limb [31,32,33].

The online adaptation network is implemented to mitigate the impact of muscle non-stationary by tuning the parameters of the other two networks. The parameters are tuned according to the reconstruction residual of sEMG signals in the autoencoder network and motion prediction error.

**Figure 3:**
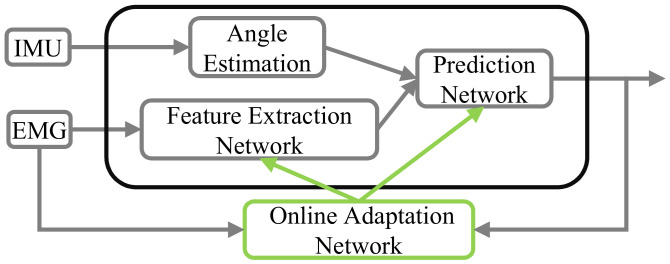
The structure of motion prediction algorithm with adaptation network.

Figure 3 presents the relationship of three networks. Those three networks are interconnected with each other. The partial input of the prediction network is the output of the feature extraction network. The adaptation network inputs are the gradients of feature extraction and prediction network, which are calculated by the sEMG reconstruction loss and motion prediction loss. The adaptation network outputs are the increment of tuned parameters of the feature extraction network and the prediction network.

#### 2.2.1. The Feature Extraction Network

**Figure 4:**
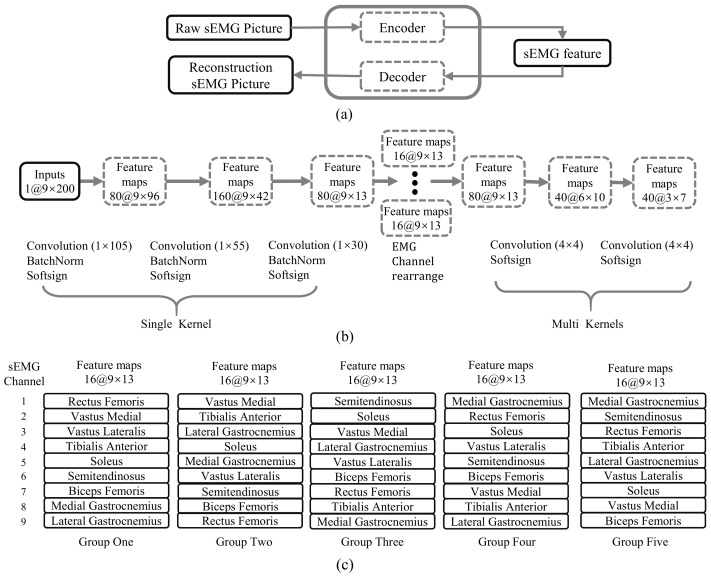
The structure of the feature extraction network. (**a**) Convolutional Auto Encoder; (**b**) The Structure of Encoder; (**c**) The rearrangement of sEMG channels.

The state-of-art research has verified the effectiveness of the convolution neural network in feature extraction of sEMG signals. Basing on the convolutional network and combining the reconstruction characteristics of the autoencoder network, this paper proposes a novel feature extraction method of sEMG signals. The feature extraction network includes two outputs: the compressive feature and the reconstructive sEMG signal. The compressive feature on behalf of the raw sEMG signals is delivered to the prediction network. The reconstructive sEMG image is compared with the input to get the reconstruction error of sEMG signals. Utilizing the reconstruction error, the encoder and decoder network parameter can be tuned to adapt to different data distributions. Figure 4 gives the architecture of the feature extraction network.

As shown in Figure 4a, the CAE can be divided into encoder and decoder parts. The two parts are symmetric with the feature output layer in the architecture. The values of corresponding parameters are different. Only the architecture of the encoder network is described in Figure 4b. The input of CAE is the sEMG image in which the horizontal axis represents the sEMG channels, and the vertical axis represents samples. The encoder part of the feature extraction network consists of five convolution layers. Those five convolution layers can be divided into the Single-Kernel part and the Multi-Kernels part. The Single-Kernel part comprises three convolution layers with one-dimensional kernel, which only compresses the information of the time domain. As presented in Figure 4b, 1×105, 1×55, and 1×30 filters with a stride of 1 and a padding of 0 have been implemented in the first three convolution layers. The different receptive field is adopted in each layer for more efficient processing of sEMG image. The Multi-Kernels part consists of two convolution layers with the 2D convolution kernel (4×4, with a stride of 1 and a padding of 0). The Single-Kernel chooses the one-dimensional convolution kernel to process the sEMG of each channel independently. In contrast, the Multi-Kernels part applies the 2D convolution kernel to mixture information from the space and time perspective. Before feeding the output of the Single-Kernel to the Multi-Kernels, the proposed method utilizes a muscle synergy trick to rearrange the sEMG channel. The muscle synergy refers to the regular relationship between the amplitude of activation and the order of activation between different muscles during movement. In the current studies, muscle cooperation is generally estimated by non-negative matrix factorization (NMF). As shown in (1), NMF divides the original sEMG matrix into two non-negative matrices. In this paper, the subscript n=200,m=9 represents the samples and channel of the sEMG signal. The subscript r=5 is a given number of synergies which is usually less than the number of channels [20,34,35,36]. NMF identifies weighted groups of muscles ($H_{r\times m}=$ synergy weights) and their activation patterns ($W_{n\times r}=$ synergy activations)

(1) $$V_{n\times m}=W_{n\times r}\cdot H_{r\times m}$$

**Figure 5:**
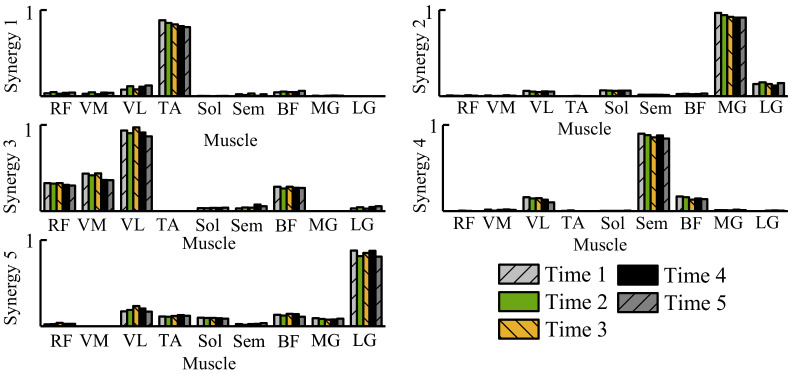
Similarity of synergy weights in different periods. Time 1∼5 represent the continuous time period, each period is 6 min of data.

For some movements, the mode of muscle activity is unique. Therefore, there is a need to consider muscle synergy when processing sEMG pictures to obtain more representative sEMG features. The proposed method rearranges the horizontal axis of the sEMG image (representing different muscles) to incorporate muscle synergy into the feature extraction network architecture. When two sEMG channels with a clear pattern are relatively far apart in the sEMG image, the traditional convolutional network needs to undergo multiple layers with two-dimensional kernel to express this cooperative relationship due to the limitations of the convolution kernel. The rearrangement by muscle synergy means cooperative sEMG channels should be as close as possible or coincide within a convolution kernel. It may only need one layer to get the pattern of the two muscles. Figure 5 gives the coordination between different muscle channels estimated by NMF. As shown in Figure 4b, 1 sEMG image with the size of 9×200 is converted into 80 feature maps with the size of 9×13 after the process of the Single-Kernel part. Then, those 80 feature maps are divided equally into 5 groups. The horizontal axis of feature maps (different sEMG channels) for each group is rearranged, as Figure 4c shown. After rearrangement, the five groups of feature maps are combined and transmitted into the Multi-Kernels part.

#### 2.2.2. The Prediction Network

The primary function of the prediction network is to predict the future motion of human. Considering the periodicity of lower limb movement, the proposed algorithm implements the long short-term memory (LSTM) [37,38] to accomplish the goal. The prediction network consists of two independent parts corresponding to the angle and event prediction. Both of them implement the same LSTM architecture as the prediction method, however, with different parameters setting.

**Figure 6:**
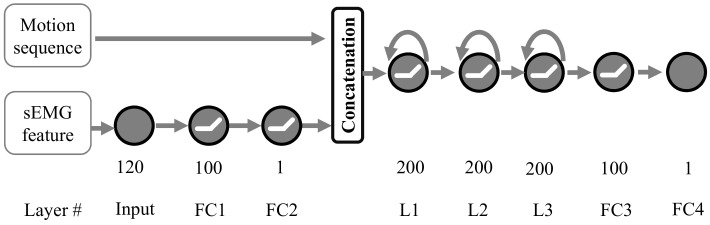
The architecture of motion prediction network.

Figure 6 shows the architecture of the motion prediction network. The input of the network consists of motion sequence and sEMG feature. The motion sequence with the size of 7×5 contains current and historical joint angle, angular velocity, and acceleration of thigh and shank. Seven represents the time, and five means the number of features. The knee joint angle is estimated using our proposed method based on the projection of gravitational acceleration in two local adjacent IMU coordinates. Despite existing errors, the IMU-based joint angle estimation method is still superior in the exoskeleton control system. This paper focuses on motion prediction and does not consider the angle error caused by the IMU estimation method. The acceleration and angular velocity of the thigh and shank are estimated using the IMU. The sEMG feature is generated from the encoding part of the feature extraction network, as Figure 4b shows. The 40 feature maps with the size of 3×7 are converted into a 7×120 matrix.

As shown in Figure 6, the sEMG feature first feeds into two fully-connected networks (FC1 and FC2) with 100 and 1 units, respectively. The output of layer FC2 is concatenated with the motion sequence. The mixed result is delivered into three LSTM layers which contain 200 units for each layer. Finally, the other two fully connected networks (FC3 and FC4) with 100 and 1 units are used to obtain the prediction output (the future angle or time). The Relu activation function is adopted after layer FC1, FC2, and FC3.

#### 2.2.3. The Online Adaptation Tuning Network

The non-stationary on muscle means the change of sEMG distribution caused by individual differences, muscle fatigue, and the adaptation of human. Taking muscle fatigue as an example, when muscle fatigue occurs, the amplitude of sEMG will increase, and the frequency will decrease gradually [15,16,18,39]. As the change of the sEMG distribution, the motion prediction algorithm no longer meets the IID condition (independently identically distribution). The prediction accuracy and the reliability decrease with the changes. The primary function of the online adaptation tuning network is to alleviate the effect caused by the non-stationary of muscle.

The online adaptive approach is quite straightforward through tuning the parameters of the prediction algorithm. The suitable increment of model parameters can accelerate convergence and reduce shock. This paper introduces an online adaptation method [40]. The method utilizes the optimizer network to optimize the partial parameters of the optimizee network, as represented in (2). In this paper, the optimizee network is the feature extraction and prediction network. In (2), *f* represents optimizee network, and *g* is the optimizer network. The output of the optimizer network is the increment of optimizee network parameters. The optimizer network input is the gradient of the optimized parameters of feature extraction and prediction network. The ϕ is the parameters of optimizer network *g*. The θ is the parameters of the optimizee network *f*.

(2) $$\theta_{t+1}=\theta_{t}+g\left(\nabla f,\phi \right)$$

The proposed method utilizes the movement prediction error and the reconstruction error of sEMG as the objective function. The movement prediction error is one of the characteristics of predictive methods. The label of data can always be obtained automatically after a certain delay. The introduction of reconstruction error is mainly to improve the optimization efficiency of the feature extraction network. As represented in Figure 6, the feature extraction network and prediction network are connected. Considering the depth of the entire prediction and feature extraction network, the gradients of the first few layers may be small when only using the movement prediction error. Thus, the feature extraction and prediction network are detached. The loss of the prediction network does not affect the parameters of the feature extraction network. The goal of the optimizer network is to minimize the reconstruction error and the movement prediction error by tuning the parameters of the feature extraction network and prediction network, respectively.

**Figure 7:**
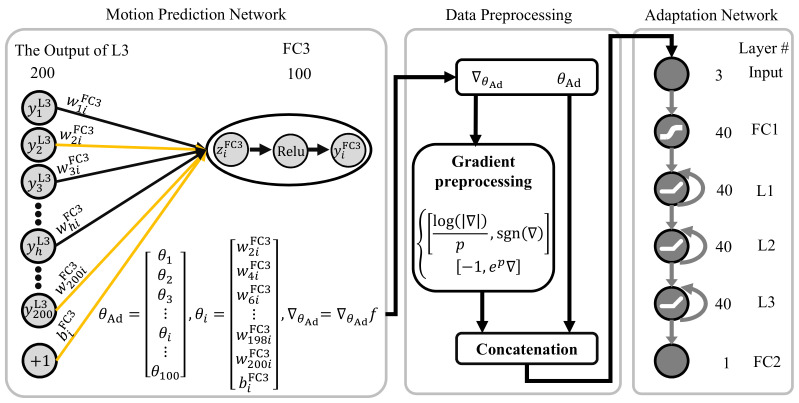
The architecture of adaptation network.

A schematic illustration of the adaptation network is available in Figure 7. The adaptation network comprises a fully connected layer FC1 with 40 units, three LSTM layers (L1, L2, L3) with 40 units, and a fully connected layer with one unit. The Tanh activation function is adopted after the first fully connected layer FC1. The adaptation network inputs are tuned parameters and its preprocessing gradient [40]. Experiments and some prior knowledge jointly determine the choice of parameters to be adjusted in the feature extraction network and prediction network. For the feature extraction network, about a quarter of the parameters of the fourth layer of the encoder are selected as the online adjustment nodes. For the prediction network, about half of the parameters of the FC3 layer are chosen as the online adjustment nodes. Figure 7 shows the process of inputs by taking the motion prediction network as an example. The FC3 layer of the prediction network is the fully connected layer that contains parameters *w* and *b* with the size of 200×100 and 100×1. The even rows of the *w* matrix (100×100) and all the *b* (100×1) are selected as the online tuned parameters. For each node in the FC3 layer, the weights and bias are first combined into the $\theta_{i}$ with the size of 101×1. Then, all the $\theta_{i}$ (i=1…100) are merged to the θ with the size of 10,100 ×1, as presented in (3) and (4). Equation (5) is the gradient of the corresponding tuned parameters. The gradients are preprocessed to scale the input’s magnitudes before feeding into the adaptation network.

(3) $$\begin{array}{c} \theta_{i}={\left[w_{2i}^{\mathrm{FC}3},w_{4i}^{\mathrm{FC}3},w_{6i}^{\mathrm{FC}3},\ldots ,w_{198i}^{\mathrm{FC}3},w_{200i}^{\mathrm{FC}3},b_{i}^{\mathrm{FC}3}\right]}^{T} \end{array}$$

(4) $$\begin{array}{c} \theta_{\mathrm{Ad}}={\left[\theta_{1},\theta_{2},\theta_{3},\ldots ,\theta_{100}\right]}^{T} \end{array}$$

(5) $$\begin{array}{c} \nabla_{\theta_{\mathrm{Ad}}}=\nabla_{\theta_{\mathrm{Ad}}}f \end{array}$$

(6) $$\begin{array}{c} \nabla =\left\{\begin{array}{ccc} \frac{\log(|\nabla |)}{p} & , & \mathrm{if}|\nabla |\geq e^{-p}\\ x_{bc} & , & \mathrm{otherwise} \end{array}\right. \end{array}$$

The output is an array (number of tuned ×1), which corresponds to the g(∇f,ϕ). The tuned parameters can be updated using the (2). The adaptation network architecture for the motion prediction and feature extraction network are the same, and the inputs are different. The input of the adaptation network for the feature extraction network is the partial parameters of the fourth layer with the size of 12,840 ×1. The fourth layer of the encoder network is the convolution layer with the 80×40×4×4 weights and 40×1 bias. Forty is the size of output, 80 is the input size, and 4×4 is the size of the filter kernel. Similar to the prediction network, all the bias and the index divisible by 4 of the weights’ first dimension are selected as the online tuned parameters. The tuning processing of the motion prediction network and feature extraction network are independent. The prediction network parameters are adapted using the motion prediction error, while the parameters of CAE are tuned according to the reconstruction error of the sEMG image.

### 2.3. Experiments

We evaluated our approach using the collected database. The goals of the experiment are to verify the prediction performance and the effectiveness of the adaptive algorithm dealing with the non-stationary on muscle. The experiments are designed to prove the effectiveness of each part.

The collected walking data are divided into two groups using the leave-one-subject-out method. Group A consists of nine subjects used to train and test the motion prediction algorithm. Group B contains only one subject for testing the effect of non-stationary on motion prediction algorithm. Therefore, we need to do 10 experiments corresponding to the 10 subjects contained in group B. For each experiment, all the parameters of the neural network are initialized randomly.

Since the three networks are not independent of each other, the training processes of the feature extraction network, the prediction network, and the adaptive network were carried out in turn. When training the feature extraction network, the data of 9 subjects in group A were shuffled with the 10-fold cross-validation method. We used the stochastic gradient descent (SGD) with a batch size of 100, an epoch number of 340 for all experiments. The learning rate was set to 0.01 and was multiplied by 0.8 when the epoch was divisible by 40. After the feature extraction network was trained, the parameters of the encoder and decoder network were fixed. Then, the prediction network can be trained. The sEMG images from shuffled group A were first fed into the encoder of trained feature extraction network to obtain the sEMG features. The sEMG features combining with the motion sequences were delivered to the motion prediction network. The parameters of the motion prediction network were updated according to the prediction error. The stochastic gradient descent (SGD) with a batch size of 100 was employed. The default learning rate was set to 0.1 and was multiplied by 0.5 when the epoch was divisible by 40. When training the motion prediction network, the batch size, learning rate, and learning rate decay for the five labels (time to heel strike, joint angle after 6.75 ms, 33.75 ms, 67.5 ms, and 101.25 ms) were the same. Moreover, the training process was independent. Two types of testing experiments for the feature extraction network and the prediction network had been conducted: inter-subject test and intra-subject test. Since groups A and B are not crossed, the individual differences of sEMG and the sEMG distribution of the two groups were different. The intra-subject test was used to verify the effectiveness of the feature extraction and prediction network. The inter-subject test was used to illustrate the robustness of the algorithm and the necessity of adaptation.

The training process of the online adaptation network is somewhat complicated and fully coupled with another two networks. Before training the online adaptation network, the feature extraction and the prediction network were trained using the shuffled group A and the parameters were fixed with the trained value. When training the online adaptation network, the data of group A were shuffled according to the subject. In each epoch training, the shuffled subject data were first delivered to the feature extraction and the prediction network to obtain the gradient of parameters to be adjusted. The gradients were passed to the online adaptation network in order to get the increment of parameters to be adjusted in the feature extraction and prediction network. Then, the parameters to be adjusted in the feature extraction and prediction network were updated. Eventually, the online adaptation network was updated according to the prediction and reconstruction error calculated by the new extraction and prediction network. The parameters chosen to be adjusted of trained feature extraction and prediction network were initialized every 10 epochs. The stochastic gradient descent (SGD) with a batch size of 100 was adopted. The epoch number was set to 550 for all experiments. The learning rate was set to 0.01 and was multiplied by 0.5 when the epoch was divisible by 100. Half of shuffled group B data were used to update the chosen parameter in the feature extraction and prediction network when testing the online adaptation network. Group B data were first transmitted to the trained feature extraction and prediction network to obtain the gradient of chosen parameters in those networks. Moreover, the gradients were delivered to the trained online adaptation network to obtain the increment. The chosen parameters to be adjusted in trained feature extraction and prediction network eventually were updated by those increment. After each iteration, the other half of the data were used to test the adaptive effect. All training and testing procedures were completed on a workstation with one Nvidia 1080Ti (CA, USA).

In this paper, two comparative experiments were designed to evaluate the influence of muscle synergy trick on the feature extraction network. One was with random rearrangement, and the other was without. Two comparative experiments (different layers, different parameters for the same layer) on online adaptation network were also conducted to evaluate the influence of chosen optimized parameters on the online adaptation algorithm. The proposed adaptation method was used to adjust the parameters of even index in the FC3 layer of the prediction network. The comparative experiment of different layers means that the adaptation method adjusts all parameters of the FC4 layer of the prediction network. The comparative experiment of different parameters in the same layer means that the adaptive method adjusts all the parameters selected in the FC3 layer of the prediction network.

For exoskeleton experiment, the collected sEMG and IMU data were not used to train the prediction and are only used to test network performance. The exoskeleton data were processed and labeled as the data without the exoskeleton. The data with assistance and without assistance were adopted to test the prediction performance, respectively. To verify the effect of the adaptive algorithm, half shuffled assistance data were used to update the parameters of the prediction and feature extraction network according to the online adaptation network. The other half of shuffled assistance data were used to test the performance of adaptation.

All time-trajectories of interest were separated into strides using the FSR. In motion prediction, the maximum prediction error is adopted to evaluate the effect. In the feature extraction experiment, the total variance (tVAF) of each sEMG image was introduced to represent the performance of sEMG reconstruction. The tVAF is defined as:

(7) $$tVAF=1-\frac{SSE}{SST}=1-\frac{∥EMG-EMG_{Re}{∥}^{2}}{{∥EMG∥}^{2}}$$

which compares the sum of squared errors (SSE) to the total squared sum of the sEMG data (SST) for each image.

## 3. Results

The motion prediction system consists of feature extraction, motion prediction, and sEMG online adaptation. The goal of the motion prediction system is to obtain the reliable and precise human motion intention. This paper verifies the performance of each part separately.

### 3.1. Feature Extraction and Adaptation

The goal of feature extraction is to obtain the compressed sEMG feature. The sEMG reconstruction error is used to quantify the non-stationary on muscle and tunes the model parameters.

**Figure 8:**
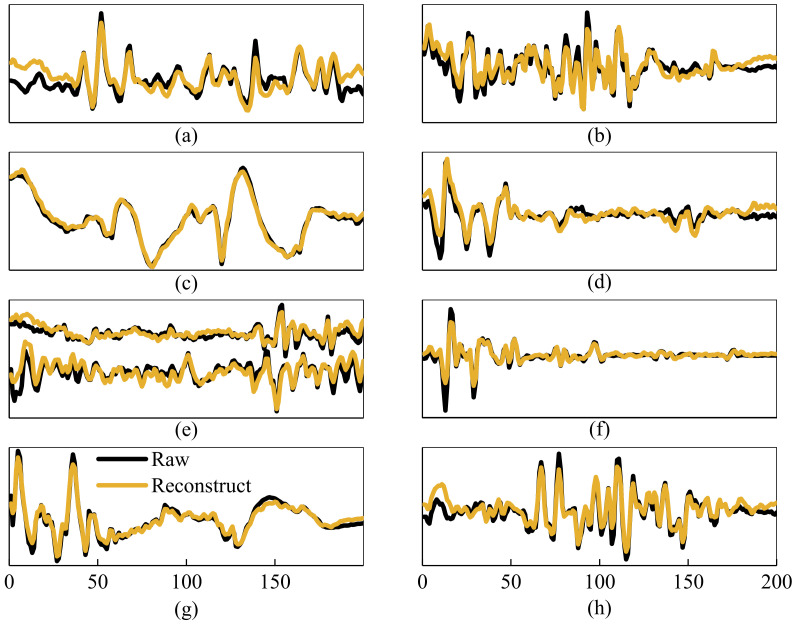
The raw and the reconstruction sEMG signals of the nine muscles. On the horizontal axis is time and on the vertical axis is myoelectric amplitude. (**a**) Rectus Femoris; (**b**) Vastus Medial; (**c**) Vastus Lateralis; (**d**) Tibialis Anterior; (**e**) Soleus and Semitendinosus; (**f**) Biceps Femoris; (**g**) Medial Gastrocnemius; (**h**) Lateral Gastrocnemius.

Figure 8 gives the results of the intra-subject test of the feature extraction network. The yellow curve represents the reconstructed sEMG signals, and the black curve represents the raw signals for each channel. The tVAF can achieve 90.71±3.42% for intra-subject testing. The reconstructed sEMG signals almost coincide with the raw sEMG signals, which indicates that the sEMG feature compressed by the encoder contains almost all the information of the original sEMG signal. Although the reconstructed signal does not coincide exactly with the raw sEMG signals in some details, such as the initial time of Figure 8a, the trend is the same.

**Figure 9:**
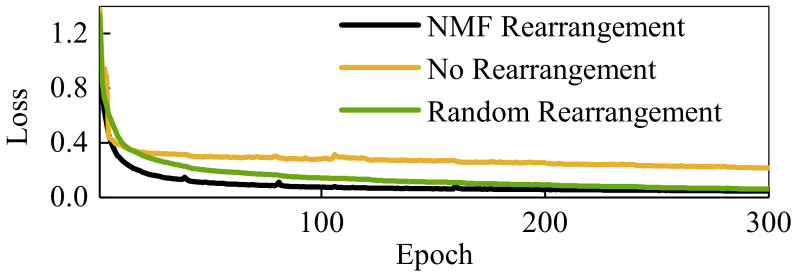
Changes of loss with proposed and two comparative convolutional autoencoder architectures. NMF rearrangement means the muscle channel of the sEMG image is rearranged according to the muscle synergy trick. No rearrangement means the muscle channel of the sEMG image does not rearrange. The random rearrangement means the muscle channel of the sEMG image is rearranged but rearrangement random.

Figure 9 gives the change of loss function for three CAE architectures. The more smaller the reconstruction error is, the more the features after dimension reduction can represent the raw sEMG image. Rearranging sEMG signal channels in the encoder of CAE can achieve a faster convergence rate and smaller reconstruction error. After 300 rounds of training, the CAE architecture with no rearrangement still cannot fully reconstruct the original sEMG signal. However, there is still a downward trend in the architecture without rearrangement.

As substantiated in our previous work [29], the size and coordination of the convolutional kernel directly affect the efficiency of the feature extraction network. In addition, the sequence of sEMG channels for sEMG image also affects the performance of the algorithm. The results indicate that mixing channels according to the results of muscle synergy can enable the network to learn the coordination relationship between muscles more quickly and accelerate the convergence speed of the convolution autoencoder network. Without rearrangement, the CAE needs to go through multiple layers of convolution to represent this coordination. Therefore, the CAE without rearrangement needs more time to convergence than CAE with rearrangement, as shown in Figure 9.

### 3.2. Motion Prediction and Adaptation

**Figure 10:**
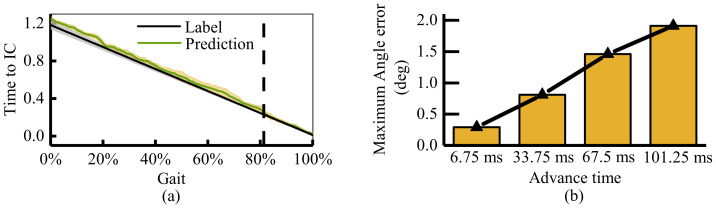
The results of motion prediction. (**a**) The results of predicted time to heel strike. The green curve is the prediction result, and the black curve is the actual time to hell strike. (**b**) The mean of maximum angle error for four groups of joint trajectory prediction.

Figure 10a provides the results of the predicted time to initial contact. It compared the mean and the standard deviation of the label and predicted time. The black line and gray shadow represent the mean and standard deviation of real-time to initial contact. Considering the speed difference of each step, the time for each gait is not the same. In terms of the standard deviation, this gait time uncertainty indicates that the standard deviation is larger at the beginning of the gait, as shown in Figure 10a.

The green line and yellow shadow in Figure 10a represent the mean and the standard deviation of predicted time to initial contact, respectively. At the beginning of the gait, the prediction error is large. Over time, the predicted value approaches the true value gradually until about 82% of the gait cycle is almost identical (The error is less than 6 ms). At the beginning of the gait, due to the uncertain sensitivity of the motion and the irregular noise of the IMU sensor, the prediction results are more uncertain. The closer the endpoint is, the less the motion uncertainty (the gray shadow in Figure 10a) is, and the more accurate the prediction results are. Ultimately, the prediction algorithm can predict the time to IC 236±9 ms at least in advance.

**Figure 11:**
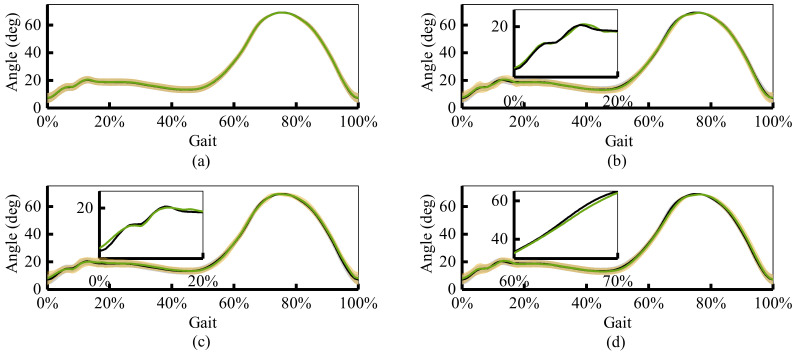
The mean and standard deviation results of four groups of joint trajectory prediction label. The green curve is the prediction result of joint trajectory. The black curve is the actual joint trajectory. All the results are drawn in a gait stride. (**a**) Prediction of joint angle 6.75 ms ahead of time; (**b**) Prediction of joint angle 33.75 ms ahead of time; (**c**) Prediction of joint angle 67.5 ms ahead of time; (**d**) Prediction of joint angle 101.25 ms ahead of time.

Figure 10b and Figure 11 present the results of angle prediction. Different from the event prediction, which predicts the occurrence time for the specific event, angle prediction is the process of predicting future angle changes at a fixed time in advance. If the horizontal axis is the absolute time, the green curve will be in front of the black curve. That is, the predicted results are prior to the actual occurrence. Figure 11 shows the angle prediction results at 6.75 ms, 33.75 ms, 67.5 ms, and 101.25 ms advanced time. It provides the mean and the standard deviation results of prediction and label data for each gait. The picture-in-picture is the maximum error for each experiment. Figure 10b provides the mean of maximum error for each gait.

As represented in Figure 10b, with the increase of the advanced time, the accuracy of prediction decreases gradually. The mean of maximum angle errors for four groups angle label are $0.29^{\circ },0.81^{\circ },1.46^{\circ },$ and $1.91^{\circ }$. When the predicted time is less than 33.75 ms (5 IMU sampling intervals), the proposed algorithm can predict the joint angle change almost exactly. When the prediction time reaches 101.25 ms, the prediction accuracy decreases obviously, the maximum prediction error is around two degrees. The error generally occurs at two time periods: 0∼20% and 60∼70%. For the first period, it is mainly caused by the uncertainty of IMU. For the foot heel strike, the impact force vibrates the skin. This uncertain skin vibration increases the complexity of angular velocity and acceleration signals. It decreases the accuracy of joint angle estimation. The standard deviation of the label trajectory shows this uncertainty. As for the second period, it mainly occurs at the prediction of the joint angle 101.25 ms ahead of time. Too long advanced prediction time and angle change speed also lead to the decline of the prediction effect.

### 3.3. Online Adaptation

The online adaptive mechanism is mainly to ensure the prediction accuracy of the algorithm when the data distribution shifts. In practice, the variation of sEMG signal and individual difference of motion will lead to the deviation of the final online prediction results. In this paper, we have designed the online adaptation algorithm for the feature extraction network and motion prediction network, respectively.

**Figure 12:**
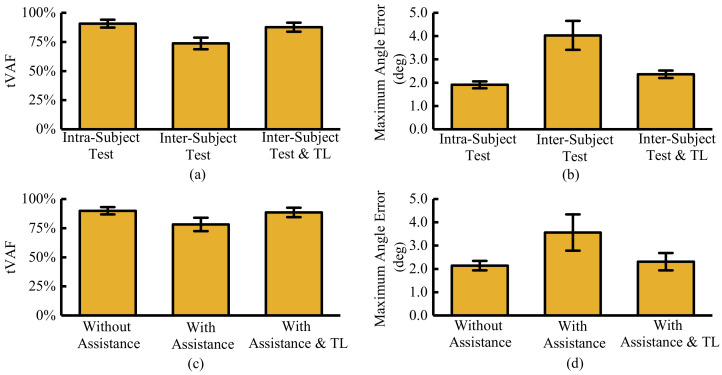
The result adaptation of feature extraction and motion prediction network with and without exoskeleton. (**a**) The adaptation results of CAE; (**b**) The adaptation results of motion prediction; (**c**) The adaptation results of CAE with exoskeleton; (**d**) The adaptation results of motion prediction with exoskeleton. ‘Inter-subject test & TL’ means the feature extraction and prediction network are trained using data of group A and tested using the data from group B. Meanwhile, the partial parameters of feature extraction and prediction network are online tuned by the adaptation network.

Figure 12 shows the adaptation results of the feature extraction and the motion prediction network with and without the exoskeleton. Group A and group B are the sEMG data from different individuals whose distributions are not the same. The intra-subject test uses the data from group A to train and test feature extraction and prediction network. The inter-subject test uses the data from group A to train the feature extraction and prediction network. We also use the data from group B to test the feature extraction and prediction network. With the same data distribution of the test set and training set, the reconstruction accuracy of EMG signal (tVAF) can reach 90.71±3.42%, as ‘intra-subject test’ represented in Figure 12a. When distribution changes (different individuals), the tVAF quickly dropped to 73.7±5.01%, as ‘inter-subject test’. Without adaptation, the reconstruction accuracy decreased from 90.71±3.42% to 73.7±5.01%. On the one hand, it proves that the change of data distribution does affect the effectiveness of the algorithm. On the other hand, it is also proved that the convolutional network has certain robustness in processing sEMG signals. The ‘intra-subject test & TL’ means the changes in distribution and the parameters are tuned by the online adaptation network. After tuning, the tVAF raises to 87.65±3.83%. The tVAF with online adaptation is slightly lower than the identical distribution results, but it still meets the demand. The same phenomenon also occurs in the motion prediction network. As represented in Figure 12b, without adaptation, the maximum prediction angle error of 101.25 ms advanced time raises to $4.03\pm 0.62^{\circ }$ from $1.91\pm 0.15^{\circ }$. The mean and the standard deviation have obvious changes. After adaptation, the maximum angle error drops to $2.36\pm 0.15^{\circ }$. Although it does not reach accuracy without distribution deviation, it has been greatly improved. In this paper, we use the relative growth rate of accuracy ($\frac{{\mathrm{Accu}}_{\mathrm{Inter}-\mathrm{Sub}\&\mathrm{TL}\;}-{\mathrm{Accu}}_{\mathrm{Inter}-\mathrm{Sub}}}{{\mathrm{Accu}}_{\mathrm{Inter}-\mathrm{Sub}\&\mathrm{TL}\;}-{\mathrm{Accu}}_{\mathrm{Intra}-\mathrm{Sub}}}$) to describe the adaptation performance. The proposed method can achieve 78.77% relative growth rate. The AdaBN method used by Du Yu et al. [27] can achieve 57.48% relative growth rate of inter-session evaluation. The relative growth rate of inter-subject evaluation only reaches 27.35%. Compared with AdaBN, our proposed method is a supervised transfer learning method. The characteristic of prediction and reconstruction algorithms is that it can be self-labeled. Those supervised transfer learning can improve the efficiency of data.

Figure 12c,d show the influence of exoskeleton on the prediction algorithm and feature extraction algorithm with assistance and without assistance. In the case of wearing an exoskeleton, even without assistance, the weight of the exoskeleton affects the movement and muscle activation. As represented in Figure 12c, the tVAF with closed exoskeleton is 89.92±3.11%. When the exoskeleton turns on, the tVAF quickly dropped to 78.21±5.78%, as ‘with assistance’ shows. The assistance provided by the exoskeleton directly affects the distribution of sEMG signals. Meanwhile, considering the interaction forces of each gait cycle are not exactly the same, the uncertain of tVAF is larger than in other cases. The ‘With Assistance & TL’ means the prediction and feature extraction network are partially updated using the half shuffled sEMG data with assistance. After the adaptation, the tVAF raises to 88.43±4.05%. The same phenomenon also occurs in the motion prediction network with the exoskeleton. With the closed exoskeleton, the maximum angle error reaches $2.14\pm 0.2^{\circ }$. When the exoskeleton turns on, the maximum prediction angle error of 101.25 ms advanced time raises to $3.56\pm 0.78^{\circ }$. On the one hand, the interaction forces change the sEMG and affect the performance of the feature extraction network. The extracted sEMG feature with assistance cannot completely characterize the raw sEMG. The variable sEMG features affect the performance of prediction. On the other hand, the interaction forces directly change the joint angle. After adaptation, the maximum angle error drops to $2.31\pm 0.37^{\circ }$. The assistance of the exoskeleton has a direct impact on the performance of the prediction algorithm and feature extraction network. The accuracy of the two networks decreases, obviously. However, the proposed adaptation network can effectively suppress the influence of exoskeleton assistance.

**Figure 13:**
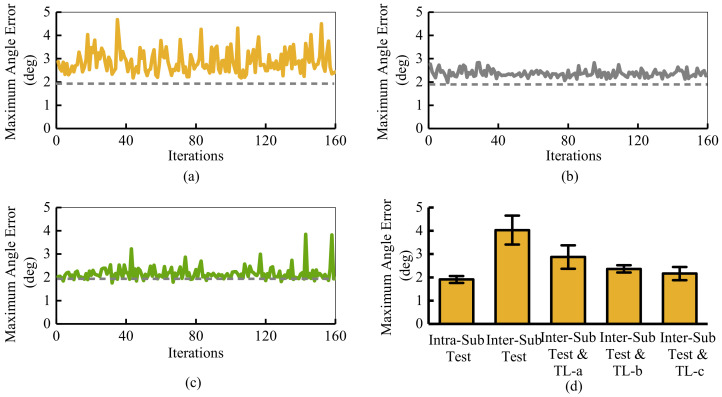
The 101.25 ms advanced motion prediction and adaptation results for two comparative experiments on online adaptation network. (**a**) Adjusting the parameters of FC3 layer; (**b**) Adjusting the parameters with even index of FC3 layer; (**c**) Adjusting the parameters of FC4 layer; (**d**) The adaptation results of angle prediction. Different parameters chosen to be adjusted are in the motion prediction network. The ‘-a’,‘-b’, and ‘-c’ in sub-figure (**d**) represent the adaptation network adjusts all the parameters of the FC3 layer, the parameters of even index in the FC3 layer, and the parameter in the F4 layer, respectively.

The comparative experiments reveal the influence of chosen optimized parameters on adaptation performance more intuitively. As shown in Figure 13d, without any adaptation, the accuracy of 101.25 ms advanced motion prediction decreases rapidly from $1.91^{\circ }$ to $4.03^{\circ }$, when the data distribution changes. Figure 13 also compares the results of three online adaptation methods with different adjusted parameters.

Figure 13a–c describes three groups of results for adjusting different parameters. The horizontal axis of Figure 13a–c is the number of iterations. The vertical axis represents the maximum angle prediction error after each iteration. All three experiments belong to the intra-subject testing. Figure 13a adjusts all parameters of the FC3 layer in the prediction network. The maximum angle error reaches the 2.88±0.50 deg. Figure 13b is the proposed architecture only tuning half of the parameters of the FC3 layer. The maximum angle error decreases to 2.36±0.15 deg. Figure 13c is the result of adjusting the parameters of the FC4 layer. The maximum angle error decreases to 2.16±0.28 deg. No matter which parameters are adjusted, the online adaptive algorithm can greatly improve the prediction accuracy. Moreover, optimization is fast and requires only one iteration.

In some details, the adaptation performance is directly influenced by the chosen parameters to be adjusted. As presented in Figure 13c, the highest prediction accuracy can be obtained by adjusting the last layer (FC4). Its accuracy can even exceed the data whose distribution does not change. However, the closer the adjusted parameters are to the output layer, the more sensitive the prediction accuracy is to the minor variation of parameters. As described in Figure 13c, the maximum prediction angle error suddenly drops to $3.85^{\circ }$. This sudden fluctuation is not conducive to the exoskeleton system. In addition, the number of adjusted parameters also affects the effect of online adaptation. As represented in Figure 13a,b, the more parameters, the lower the accuracy and the worse the stability. Thus, the deeper the layers of the adjusted parameters are, the higher the classification accuracy is to be achieved. Meanwhile, the fewer the parameters are to be adjusted, the higher the accuracy is to be achieved.

## 4. Discussion

In this work, an online adaptation human intention prediction method was proposed. The proposed algorithm can effectively predict human motion in advance to reduce the coupling between human and machine systems. In exoskeleton control, angle and special event are two commonly used variables. As the high-level control, the angle signal can be mapped to the motion phase. Then the corresponding control strategy can be made according to the phase. The angle signal can also be used as the input of the dynamic model to calculate the joint torque. As the low-level control, the angle signal can be used as feedforward to compensate the active motion of the human joint in the process of force loading. The prediction results in this paper can meet the requirements of these conventional applications. At the same time, it can realize the prediction in advance. In the real exoskeleton, the most common strategy is to plan the assistance strategy of a gait cycle in advance and then detect a special event (such as heel strike) and apply force according to the defined strategy. The detection of the special event is critical. When the detection of this special event is delayed, there will be a deviation between the assumed motion and the real motion during the whole gait cycle, which will result in the decline of assistance performance. Predicting motion in advance means that the high-level control system can transmit force commands to the low-level control in advance before the actual heel strike. Appropriate advances can effectively reduce the force tracking performance degradation caused by mechanical and acquisition delay.

The proposed prediction algorithm not only ensures the accuracy of prediction but also ensures the reliability of the algorithm. Even if the data distribution changes, the proposed algorithm can still guarantee performance. In practical application, the algorithm can effectively resist the disturbance of muscle fatigue, individual differences, and individual adaptation. The experiment verifies the effectiveness of the algorithm in collected data. The limitation of this paper is the lack of more complex motion data, despite that the walking speed of collected data is variable. Comparing with normal walking in daily life, the data of treadmill walking is relatively smooth. In future work, we plan to focus on complex motions, such as different speeds, motion patterns, and different ground conditions. Moreover, the effect of different interaction forces and assistive time on algorithm are also the focus of further research.

## 5. Conclusions

In this paper, we proposed a real-time human intention prediction method utilizing the sEMG and IMU signals which can achieve motion prediction with high accuracy and reliability.

The proposed method can predict heel strike 236±9 ms in advance and knee joint angle at least 101.25 ms in advance, respectively. In order to ensure the reliability of the prediction results, we propose a hybrid feature extraction network and online adaptation network to deal with the complex non-stationary sEMG signals. The feature extraction network, constructed by the convolution autoencoder network, is imported to mining the motion information hidden in the sEMG signals and to assist the motion prediction. The online adaptation network is introduced to mitigate the impact of changes in data distribution in online applications. By online monitoring and adjusting the parameters of feature extraction and prediction network, the online adaptation network can make the motion prediction algorithm adapt to the change of data distribution.

## Data Availability

Data sharing not applicable.
